# *Plasmodium vivax* Infections among Immigrants from China Traveling to the United States 

**DOI:** 10.3201/eid3007.240177

**Published:** 2024-07

**Authors:** Paloma Khamly, Nahel Kapadia, Minette Umali-Wilcox, Susan M. Butler-Wu, Kusha Davar

**Affiliations:** Los Angeles General Medical Center, Los Angeles, California, USA (P. Khamly, N. Kapadia, M. Umali-Wilcox, S.M. Butler-Wu, K. Davar);; Keck School of Medicine of University of Southern California/Los Angeles, Los Angeles (S.M. Butler-Wu)

**Keywords:** Malaria, Plasmodium vivax, Anopheles mosquitoes, vector-borne infections, zoonoses, parasites, emigration and immigration, hospitals, China, Ecuador, Mexico, Panama, United States

## Abstract

Beginning in 2023, we observed increased *Plasmodium vivax* malaria cases at an institution in Los Angeles, California, USA. Most cases were among migrants from China who traveled to the United States through South and Central America. US clinicians should be aware of possible *P. vivax* malaria among immigrants from China.

*Plasmodium vivax*, the most widely geographically distributed species of the *Plasmodium* genus, causes malaria in humans and is transmitted through the bite of infectious *Anopheles* mosquitoes. *P. vivax* is the second most prevalent cause of malaria globally and constitutes a large portion of the annual malaria cases in the Western Hemisphere; ≈397,000 cases of *P. vivax* malaria were reported in the Americas in 2022 (*1*). Conversely, *P. vivax* malaria is relatively infrequently encountered at most institutions in the United States because most cases are travel-associated. The Centers for Disease Control and Prevention (CDC) reported 72% of all *P. vivax* cases in the United States in 2018 were imported from malaria-endemic countries (*2*). A central epidemiologic factor of *P. vivax* is its ability to establish a dormant liver stage that can later reactivate, leading to episodic parasitemia. This latent stage poses a potential risk for transmission to another human through a mosquito vector if appropriate treatment is not administered (*3*).

Since early 2023, Los Angeles General Medical Center in Los Angeles, California, USA, has observed a concerning rise in *P. vivax* cases, specifically among immigrants from China entering the United States via the southern US border. We diagnosed 10 cases of *P. vivax* malaria, 9 of which were among immigrants from China who came to the United States by land via South and Central America. In contrast, we only saw 2 cases of *P. vivax* at our institution during 2016–2022, one patient in 2017 and another in 2018, neither of whom were of Asian descent. In addition, we saw 1 case of non­–*P. vivax* malaria during that timeframe. All cases were diagnosed by thick and thin blood smear microscopy and the BinaxNOW Malaria test (Abbott Laboratories, https://www.abbott.com).

Whether any of the 9 immigrants from China traveled together is unknown because they sought care individually at our institution. They all met criteria for uncomplicated malaria and were treated with either hydroxychloroquine, chloroquine, or atovaquone/proguanil, followed by antirelapse treatment with primaquine (Table). Upon further correspondence with nearby microbiology laboratory directors, similar findings of dramatic increases in *P. vivax* cases since 2023 have also been observed in at least 1 local hospital that serves as a catchment area in the San Gabriel Valley, California, with a majority Asian American population. All cases were acquired by travel, and we noted no evidence of local transmission.

**Table:** Characteristics and treatment regimens of patients with diagnosed *Plasmodium vivax* infections among immigrants from China traveling to the United States via Central and South America, Los Angeles, California, USA, January 2023–April 2024*

Case no.	Date diagnosed	Clinical signs and symptoms	Duration of symptoms	% Parasitemia	Outpatient vs. inpatient	Treatment	Antirelapse treatment
Case 1	Mar 2023	Fever, chills, myalgias, shortness of breath	2 mo	0.10%	Inpatient	HCQ	Primaquine
Case 2	Mar 2023	Fever, diaphoresis, nausea	1.5 mo	0.30%	Inpatient	AP	Primaquine
Case 3	May 2023	Fever, fatigue, myalgias	9 d	<0.20%	Outpatient	AP	Primaquine
Case 4	Jun 2023	Fever, chills	1 wk	0.13%	Outpatient	Chloroquine	Primaquine
Case 5	Sept 2023	Fever, headache, diarrhea, nausea, vomiting	1 wk	0.05%	Outpatient	AP	Primaquine
Case 6	Jan 2024	Fever, cough	10 d	<0.20%	Outpatient	AP	Primaquine
Case 7	Jan 2024	Fever, chills, myalgias, nausea	10 d	<0.20%	Inpatient	AP	Primaquine
Case 8	Jan 2024	Fever, chills, myalgias	10 d	0.20%–1.0%	Inpatient	AP	Primaquine
Case 9	Jan 2024	Fever, chills, fatigue, cough	4 d	0.20%–1.0%	Inpatient	Chloroquine	Primaquine

*All patients met criteria for uncomplicated malaria. AP, atovaquone/proguanil; HCQ, hydroxychloroquine.

Of note, the United States Border Patrol reported a 1,000% increase in the number of immigrants from China arriving at the southern border during 2023 compared with previous years (*4*). The immigrants are primarily following a well-traveled route that begins in Ecuador, a country that does not require visas for citizens of China. From there, they traverse the jungle terrain of Panama’s Darién Gap, proceeding into Central America and Mexico before arriving at the southern US border.

Hospitals serving newly arrived immigrants should be cognizant of this new emigration route from China via South and Central America and the associated risk of acquiring *P. vivax* malaria. All patients should be screened for malaria when they have compatible symptoms, and a detailed travel history should always be obtained. A vital detail to consider with travel history is that patients with prior *P. vivax* infection can relapse weeks, months, or years after initial diagnosis because the parasites can lay dormant in the liver as hypnozoites (*5*). Persons with diagnosed malaria should be assessed for severe symptoms, such as impaired consciousness, severe anemia, acute kidney injury, acute respiratory distress, or shock. For severe *P. vivax* malaria, patients typically are treated with intravenous artesunate. For uncomplicated *P. vivax* malaria, providers can prescribe chloroquine, hydroxychloroquine, artemether/lumefantrine, or atovaquone/proguanil, depending on endemic country-specific resistance factors and institutional formulary supply. Primaquine or tafenoquine are used afterwards as antirelapse treatment. Full treatment recommendations can be found on the CDC website (*6*). In addition, the CDC malaria hotline provides for immediate assistance (*7*).

Of note, China was declared malaria-free by the World Health Organization in 2021, and no indigenous cases of malaria had been reported since 2016, suggesting that travel from China is not an epidemiologic risk factor itself (*8*). If feasible, persons embarking on travel via the South and Central America route should consider taking malaria prophylaxis. 

In conclusion, clinical microbiology laboratories, particularly those in border states, should consider implementing rapid antigen testing for malaria to improve turnaround time for case detection but should be aware of the potential for false-negative results in patients with low parasitemia levels (*9*). Clinicians also should be aware of the possibility for an increase in *P. vivax* malaria cases among immigrants from China arriving via the southern US border.
